# Developing guidelines on EFT for same‐sex/gender relationships: Recommendations from a Delphi study

**DOI:** 10.1111/famp.13079

**Published:** 2024-11-10

**Authors:** Caitlin Edwards, Andrea K. Wittenborn, Robert Allan

**Affiliations:** ^1^ Human Development and Family Studies Colorado State University Fort Collins Colorado USA; ^2^ Psychiatry and Behavioral Medicine Michigan State University Grand Rapids Michigan USA; ^3^ University of Roehampton London UK

**Keywords:** cultural adaptation, Delphi method, emotionally focused therapy, same‐sex and same‐gender relationships

## Abstract

Emotionally focused therapy (EFT) is one of few empirically supported treatments for relationship distress. While evidence‐based approaches are critical for ensuring safe and effective treatment, EFT has not been adapted for use with same‐sex/same‐gender (SS/SG) relationships. This study used the Delphi method to generate consensus on treatment guidelines for using EFT with SS/SG relationships. Forty therapists with clinical expertise in EFT for SS/SG relationships were recruited. Data were collected in three phases. In phase one, participants responded to open‐ended questions regarding how EFT should be adapted for SS/SG relationships. Phases two and three involved participants rating the importance of the recommended guidelines. Data were analyzed using thematic analysis and descriptive statistics. The final recommendations included 49 guidelines on EFT for SS/SG relationships. The data reflected modifications to therapists' foundational knowledge and development, practice set up and orientation, and the three stages and nine steps of EFT.

Research demonstrates that culturally adapted therapy is more effective than nonadapted therapy (Nagayama‐Hall et al., 2016; Soto et al., 2018). While emotionally focused therapy (EFT; Johnson, 2019; Johnson & Greenberg, 1985) is an empirically supported treatment with a substantial research base (Spengler et al., 2024), to date, there has been no empirical research on the application of EFT to lesbian, gay, bisexual, transgender, or queer/questioning (LGBTQ+) relationships or members of the LGBTQ+ communities in same‐sex/same‐gender (SS/SG) relationships. Therefore, this Delphi study provides the first empirical findings on therapists' recommendations for the adaptation of EFT with SS/SG relationships.

## Emotionally focused therapy

EFT (Johnson, 2019; Johnson & Greenberg, 1985) is an empirically supported treatment for couple relationship distress (Spengler et al., 2024). EFT assumes that relationship distress results from rigid, negative interaction patterns that prevent partners from meeting each other's attachment needs (Johnson, 2019). The EFT change process (see Johnson, 2019) fosters an increasingly secure attachment bond by helping partners to identify, experience, express, and respond to attachment emotions and needs. Change in EFT occurs through a three‐stage, nine‐step process. Stage one, cycle de‐escalation and stabilization, involves alliance building and assessment (step one), identifying the negative interaction cycle (step two), accessing and expressing the previously unacknowledged attachment emotions informing each partner's position in the negative cycle (step three), and reframing the problem as the cycle, the underlying attachment emotions driving the negative cycle, and each partner's unmet attachment needs (step four). Stage two, restructuring, involves promoting identification with disowned needs, parts of self, and attachment emotions (step five), promoting each partner's acceptance of the other's experience (step six), and facilitating the expression of relational wants, needs, and attachment longings (step seven). Stage three, consolidation, facilitates the creation of new solutions to old problems (step eight) and concretizes new interactional patterns (step nine; Johnson, 2019).

## Same‐sex/same‐gender relationships

Same‐sex/same‐gender (SS/SG) relationships exist in a wider heterocentric sociocultural context that devalues LGBTQ+ relationships (Frost & Meyer, 2023), resulting in minority stress that is characterized by physical, affective, interpersonal, and intrapersonal difficulties (Frost & Meyer, 2023). Minority stress is highly correlated with a lack of commitment and investment in relationships (Oren, 2021) as well as aggression between partners (Lewis et al., 2014). This indicates SS/SG relational functioning is impaired by oppression. More specifically, SS/SG relationship functioning is negatively impacted by internalized homophobia; for same‐sex male couples, higher levels of internalized homophobia are associated with more negative relationship interactions, depression, and alcohol use (Feinstein et al., 2019). SS/SG relationship functioning is also further impacted by the level of *outness* of each partner. For example, Totenhagen et al. (2018) found lower levels of outness were associated with increased stress and lower relationship commitment. Additionally, individuals identifying as members of LGBTQ+ communities experience delays in reaching relationship‐related milestones, such as introducing a partner to one's parents (Macapagal et al., 2015).

Not only does minority stress strongly impact SS/SG relationships, but SS/SG relationships face other unique circumstances. For example, SS/SG relationships often establish relational roles that exist outside traditional gender roles that influence both labor and financial responsibilities and create family planning challenges (Scott et al., 2019). Furthermore, despite the consequences of minority stress, SS/SG relationships also demonstrate remarkable resilience, characterized by respect and appreciation of differences, positive emotions, and effective communication (Rostosky & Riggle, 2017). SS/SG relationships are also characterized by deep emotional intimacy and sexual satisfaction (Joyner et al., 2019). SS/SG relationships are often defined by different dynamics than different‐sex relationships, such as more flexible gender roles (Horne et al., 2014), more positive conflict (Gottman et al., 2003; Kurdek, 2004), and egalitarian, mutually supportive parenting (Dunne, 2013).

## Attachment theory and SS/SG relationships

Attachment theory (Ainsworth et al., 1978; Bowlby, 1969), which undergirds EFT, has been applied across several relationship types, including parent–child relationships (Mills‐Koonce et al., 2018, romantic relationships (Feeney & Fitzgerald, 2018), and friendships (Doherty & Feeney, 2004), underscoring the importance of attachment for all types of relationships. Previous research has indicated that attachment processes operate similarly for both different‐sex and SS/SG relationships (Mohr et al., 2013). However, two recent scoping reviews (Allan & Westhaver, 2018; Edwards, Allan, et al., n.d.) have recognized attachment processes as qualitatively different for SS/SG relationships compared to different‐sex relationships. For example, some attachment experiences of lesbian relationships may be more strongly influenced by the friendships formed in adolescence rather than parental relationships (Edwards, Allan, et al., n.d.) and lesbian women report higher levels of avoidant attachment (Ridge & Feeney, 1998).

## Couple interventions for SS/SG relationships

Despite the unique lived experiences of members of LGBTQ+ communities, SS/SG relationships are largely underrepresented in psychotherapy outcome research (Spengler et al., 2020). Fortunately, conceptual literature has provided resources for working with SS/SG relationships. For example, Pentel et al. (2021) provide a clinical framework for working with sexual minority relationships that address the unique lived experiences that characterize SS/SG relationships, while Addison and Coolhart (2015) describe working with SS/SG relationships from an intersectional lens. Curtis (2013) describes Gestalt couple therapy with lesbian women and Singer (2013) reviews Gestalt couple therapy with gay men. Furthermore, two couple interventions, Gottman couple therapy (Garanzini et al., 2017) and Our Relationship Program (Hatch et al., 2016), have been tested with SS/SG samples without modification.

Moreover, a recent systematic review (de Brito Silva et al., 2022) describes systemic interventions for lesbian, gay, and bisexual (LGB) clients, including culturally adapted interventions. For example, the Relationship Checkup (RC; Cordova et al., 2014) was adapted to provide affirmative care for LGBTQ + clients by modifying initial questions, revising feedback to adequately capture LGBTQ + client experiences, and adapting the manual by (a) removing heterosexist language, (b) including opportunities to discuss identity and its positive impacts, (c) discussing the impact of living in a cisheterocentric cultural context, (d) asking about support systems, and (e) including a discussion of sex and sexuality (Gray et al., 2023). Additionally, the Better Together relationship education program was specifically designed for same‐sex relationships (Whitton et al., 2018). To develop this program, researchers surveyed clinicians who worked with same‐sex relationships (Scott et al., 2019) and conducted focus groups with same‐sex couples (Scott & Rhoades, 2014). These studies, combined with extant literature, informed a modified cognitive behavioral couple therapy (CBCT) for same‐sex female couples in distress, which demonstrates strong feasibility and acceptability (Pentel et al., 2021).

However, to date, only one evidence‐based model of therapy for romantic relationship *distress* (CBCT) has been adapted to account for the unique lived experiences of same‐sex female couples (Pentel et al., 2021). This is highly relevant as research comparing culturally adapted and nonadapted interventions for specific populations found that culturally adapted therapeutic interventions are more effective than those that are not adapted (Nagayama‐Hall et al., 2016; Soto et al., 2018). Cultural adaptation involves the systematic modification of an intervention guided by a cultural adaptation framework (e.g., Bernal & Sáez‐Santiago, 2006) as well as individual tailoring to clients' backgrounds and needs (Collado et al., 2013). The systematic modification of interventions to better fit the target population's values, beliefs, goals, and needs improve engagement and clinical outcomes (Rathod et al., 2018; Steinka‐Fry et al., 2017). Therefore, therapy tailored to the unique experiences and circumstances of SS/SG relationships may be more effective and better received compared to therapy that was designed for heterosexual, cisgender (i.e., different‐sex/different‐gender) relationships (Pepping et al., 2017; Scott & Rhoades, 2014).

EFT has been applied across a variety of presenting concerns (see Wiebe & Johnson, 2016). Additionally, EFT has been conceptually applied to various populations, including clients presenting with spiritual concerns (Furrow et al., 2011), lesbian couples (Hardtke et al., 2010), and heterosexual African American couples (Nightingale et al., 2019). However, to date, only one empirical adaptation of EFT has been conducted for Japanese couples (Hattori, 2014), leaving a substantial gap in the research on EFT. Thus, systematic research that adapts EFT to account for the unique lived experiences of SS/SG relationships is needed.

## The current study

Guided by a cultural adaptation framework (e.g., Bernal & Sáez‐Santiago, 2006), this study aimed to create initial guidelines for adapting EFT for SS/SG relationships based on expert consensus. We used the Delphi method to survey expert EFT therapists who work with SS/SG relationships to gather consensus on how to modify EFT to account for the unique needs and lived experiences of SS/SG relationships. The Delphi method is a commonly used research method when little to no data exist regarding a specific topic (Keeney et al., 2011). Delphi studies aim to collect expert‐based recommendations to identify consensus on topics that have not been studied previously (Keeney et al., 2011). Specifically, the current study aimed to address the following research question: What is the expert consensus on guidelines for adapting EFT to fit the unique needs and therapeutic preferences of SS/SG relationships?

## METHOD

This study (#7442) was approved by the Michigan State University Institutional Review Board.

### Author positionality

At the time of the study, the first author was a doctoral candidate who had been mentored by both the second and third authors. Their research interests include cultural adaptation, LGBTQ+ relationships, and EFT. They identify as a White, agender, pansexual, polyamorous femme presenting person. The second author is a scholar of EFT who identifies as White, female, cisgender, and heterosexual. The third author is a gay/queer, cisgender, White male who grew up working class and is an immigrant; his research interests include exploring the impact of minority stress on queer relationships.

Both the first and third authors currently use EFT with members of LGBTQ+ communities in their respective practices. The first and third authors, as members of LGBTQ+ communities, noted that the guidelines suggested by the participants aligned with their own lived experiences as well as their own ways of practicing. Additionally, the second and third authors routinely and overtly empowered the first author throughout the research process to leverage everyone's expertise in this study. Furthermore, each of the unique skills, experiences, and contributions of the authors were routinely attended to by valuing lived experiences as a way of knowing. Consensus was routinely achieved via group meetings and discussions.

### Design

This study was guided by the Delphi method (Keeney et al., 2011). Originally developed by the RAND corporation, the Delphi method is a pragmatic approach used widely across disciplines to inform clinical practice, decision‐making, and policymaking (see Keeney et al., 2011). The Delphi method is used in both quantitative and qualitative studies (Brady, 2015), and is useful when studying topics that have limited or no empirical information. It is a helpful approach to use when other methods, such as focus groups, may result in one or more experts dominating the consensus process or when experts are located in an expansive geographic area (Keeney et al., 2011). Furthermore, the Delphi method is useful when attempting to capture the lived experiences of marginalized groups (Christie & Barela, 2005).

The Delphi method involves developing a panel of experts and gathering and analyzing data in an iterative process until consensus is achieved among participants (Diamond et al., 2014). In Delphi studies, participant consensus is defined as reaching agreement based on a predetermined cutoff (Keeney et al., 2011). We followed Diamond et al.'s (2014) recommendations to use the cutoff of 75% (i.e., 75% of participants rate a guideline as *important* or *very important*). This means that all statements rated as important or very important by 75% or more participants were accepted as recommendations. Statements rated as important or very important by 70%–74% of participants were extracted to be re‐considered for inclusion by participants. Statements rated as important or very important by 69% or fewer participants were removed from further analysis (Diamond et al., 2014).

When referring to participants in Delphi studies, the term *expert* is, by necessity, broad (Niederberger & Spranger, 2020). In this Delphi study, we used Keeney et al.'s (2011) recommendations to define an expert on EFT for SS/SG relationships. Keeney et al. (2011) indicate that *experts* in Delphi studies have been defined in several ways. For example, McKenna et al. (1994) define experts as *informed individuallls,* while Lemmer (1998) and Green et al. (1999) define expert as someone who has knowledge about a specific subject. Specifically, Keeney et al. (2011) note experts (a) have knowledge of a topic, (b) are relatively impartial, and (c) are committed to furthering knowledge about a given topic. As a result, we defined expert EFT therapists as therapists who met the following criteria: (a) completion of the International Centre for Excellence in Emotionally Focused Therapy (ICEEFT) EFT Externship and Core Skills trainings (i.e., a minimum of 78 h of training), (b) the use of EFT with SS/SG relationships for a minimum of 2 years, and (c) the use of EFT with SS/SG relationships at the time of the study (i.e., within at least the six months prior to being screened) and (d) at the time of the study, the use of EFT with more than one SS/SG relationship. This ensured that therapists had been engaged in the deliberate practice needed to develop the expertise sought from participants in this study (Chow et al., 2015; Harwell & Southwick, 2021).

When deciding upon the Delphi method, we drew on a plethora of articles that provided excellent examples of its use. Unfortunately, as the Delphi method is not frequently used in couple and family therapy (CFT) literature, we needed to draw on a broader literature. In this vein, the term “expert” appears to be interpreted broadly, as many articles (e.g., Meibos et al., 2019; Meredith et al., 2021; Mittnacht & Bulik, 2015; Taylor et al., 2020; Turner et al., 2020) use degrees and membership associations to define an *expert*. For example, Clark et al. (2022) used the following criteria to determine their expert panel: hold a membership with the Association of Child and Adolescent Counseling (ACAC) and have at least 2 years' experience counseling children and Neuer Colburn et al. (2016) recruited CACREP accredited counseling supervisors via listservs.

Specifically in reference to the use of Delphi studies in CFT, Dawson and Brucker (2001) support the use of the Delphi method in CFT research and indicate that years of clinical experience is a primary criterion for selecting the expert panel (although they do not specify how many years). Additionally, we drew on the description of the Delphi method provided by Blow and Sprenkle (2001). Blow and Sprenkle (2001) chose their panel based on exposure to a wide variety of family therapy theories, depth of clinical experience, experience in CFT, and an advanced degree in CFT. These criteria are similar to this study's expert panel, as they all have in‐depth exposure to EFT, a median number of 13.5 years of clinical experience using EFT, and a minimum of a master's degree in psychotherapy.

Per Diamond et al. (2014)'s recommendations, panelists rated their EFT expertise using a Likert scale from 1 (*minimal experience*) to 5 (*expert*) as well as their expertise in working with SS/SG relationships [1 (*minimal experience*) to 5 (*expert*)]. Participants who self‐rated 1 on EFT expertise and/or expertise with SS/SG relationships were automatically removed from the study.

### Procedures

Participants were recruited from mid‐April to mid‐May of 2022 through sampling techniques recommended for Delphi studies, including purposive (i.e., a nonrandom sampling technique that involves the deliberate choice of participants based on particular participant qualities) and snowball (i.e., a nonrandom sampling technique that involves engaging participants in identifying other potential participants) sampling methods (Fowler, 2014). The recruitment letter was shared with ICEEFT‐certified trainers, therapists, and supervisors, the general ICEEFT listserv, LGBTQ+ EFT Facebook groups, Queer EFT listserv, and professional connections. We also asked ICEEFT trainers and supervisors to distribute the recruitment letter on local EFT listservs. In addition, we searched for any books, book chapters, peer‐reviewed articles, dissertations, and conference presentations on EFT with SS/SG relationships and invited the authors to participate in the study. Finally, we invited expert nominations from all recipients of the recruitment letter, and we contacted the potential participants they recommended.

All potential participants received a description of the purpose of the study, study process, inclusion criteria, and participant expectations. Potential participants (*n* = 56) then completed a screening survey online via Qualtrics; Qualtrics automatically determined each participant's eligibility and shared the results with each participant. All participants who met the inclusion criteria enrolled in the study (*n* = 40). Potential participants met inclusion criteria if they: (a) completed the ICEEFT EFT Externship and Core Skills (or advanced) trainings (i.e., a minimum of 78 h of training), (b) had at least 2 years of post‐Externship experience using EFT with SS/SG relationships, (c) were presently engaged in using EFT with SS/SG relationships (i.e., within at least 6 months prior to screening), (d) were willing to participate in each round of data collection, (e) were over age 18, and (f) could read and write English.

It is important to minimize attrition in Delphi studies because attrition can reduce the trustworthiness of the findings. Therefore, we aimed to achieve a response rate of 70% or greater for each stage of data collection (Keeney et al., 2011). We sought to increase the response rate through reminders and compensation. Specifically, two reminders were sent 2 weeks apart to encourage survey completion for each phase. An additional reminder was sent to participants 1 week prior to the close of the survey date for phase two in order to encourage additional completion due to lower‐than‐expected participation in phase two. Participants were compensated with a $100 gift card for their participation.

### Participants

Forty participants enrolled in the study. Participants' average age was 48.72 (SD = 8.76). They were primarily White (90%) and non‐Hispanic (92.5%). They reported living in the United States (76.1%), Australia (15.2%), Canada (8.7%), or in other locations (2.2%). Exactly 70% were assigned female at birth, while 30% were assigned male. Participants were asked to choose all gender and sexual identities that applied to them. Gender identities included woman (49%), cisgender (36.7%), man (14.3%), transman (4.0%), nonbinary (4.0%), queer (2.0%), gender fluid (2.0%), prefer not to say (2.0%), and one participant wrote‐in transman/genderqueer. Participants reported their sexual orientation as heterosexual/different‐gender (45%), gay (20%), bisexual (12.5%), queer (7.5%), demisexual (2.5%), lesbian (5%), pansexual (2.5%), same gender loving (2.5%), and prefer not to say (2.5%).

Most participants' (53.2%) held a master's degree, while 42.6% held a doctoral degree, and 4.3% elected to self‐describe (e.g., Masters of Divinity). Participants had worked 3–39 years as a psychotherapist (median = 13.5 years) and all but four panelists were practicing therapy full‐time. The subjects of their highest degrees were reported as: psychology (32.5%), couple and family therapy (20%), social work (17.5%), counseling (15%), social psychology (2.5%), clinical psychology (2.5%), psychotherapy (2.5%), clinical mental health (2.5%), applied linguistics (2.5%), and interdisciplinary (2.5%).

Using the five‐point Likert scale [i.e., 1 (*minimal experience*) to 5 (*expert*)], participants rated their level of EFT expertise as three (30%), four (50%), or five (20%). Using the same Likert scale, participants also rated their level of expertise with SS/SG relationships as two (2.5%), three (50%), four (37.5%), and five (7.5%). In addition, 39 of the 40 participants also reported the percentage of SS/SG relationships that made up their caseload at the time of the study (Table 1).

**TABLE 1:** Percentage of SS/SG relationships composing participant current caseloads.

Number of participants	Percentage of the sample	Percentage of SS/SG relationships in current caseload
1	2.56%	>5%
18	46.15%	5%–10%
8	20.51%	11%–20%
5	12.82%	21%–30%
5	12.82%	41%–50%
0	0	51%–60%
0	0	61%–70%
3	7.69%	71%–80%
0	0	81%–90%
0	0	91%–100%

### Data collection

In Delphi studies, data are gathered through several phases of questionnaires that are primarily informed by participants' responses to the previous survey. In this study, three phases of data were collected in 2022: phase one from April 14 to May 25, phase two from August 3 to September 23, and phase three from November 11 to December 13. In phase one (*n* = 40), participants were asked to provide in‐depth responses to 13 questions, which asked for recommendations on how each step and task of EFT should be altered, expanded, and/or adapted when working with SS/SG relationships. Examples of questions include: (a) What guidelines are important for EFT therapists to follow when assessing and identifying negative interactional patterns with SS/SG relationships in EFT?, and (b) What guidelines are important for EFT therapists to follow when facilitating the expression of needs and wants (i.e., creating emotional engagement) with SS/SG relationships in EFT? In phase one, participants provided 215 recommendations for using EFT with SS/SG relationships.

Of the 40 original participants, 27 participated in phase two. The 215 guidelines were analyzed by the authors using Thematic Analysis (TA; Braun & Clarke, 2006, 2019) to reduce repetition and improve conciseness and clarity, which resulted in 117 guidelines. The 117 guidelines were then provided to participants in phase two. Participants were then asked to rate how important they thought each clinical guideline was using a 5‐point Likert scale [i.e., 1 (*very important*) to 5 (*very unimportant*)]. They were also given an opportunity to provide additional clinical guidelines they thought should be included. Of the initial 117 guidelines, 106 statements achieved greater than or equal to 75% consensus and were retained. Four statements achieved a consensus level between 70% and 74%; these four statements were compiled in Qualtrics and sent to participants to re‐rate in phase three. Seven statements achieved less than or equal to 69% consensus and were removed from further analysis (see Table 1). In addition, three new guidelines were recommended.

Of the 40 original participants, 29 participated in phase three. In phase three, participants were provided the four guidelines that just missed the cut‐off (i.e., were rated as important or very important by 70% to 74% of panelists in phase two) and the three new guidelines generated in phase two. They were also provided with the Likert scale rating they had selected in phase two as well as the entire sample's mean response for each guideline. They were then asked to re‐rate each guideline using the 5‐point Likert scale ranging from 1 (*very important*) to 5 (*very unimportant*). Participants who did not complete the phase two survey were still allowed to participate in phase three; however, since they did not rate the recommendations in phase two, the phase three survey they received did not include their own ratings.

The following is an example of the instructions provided to participants: “On average, participants rated this recommendation as *important*; you rated this item as *very important*. Based on this information, please rate your response to the recommendation below using the 5‐point scale: Understand LGBTQ+ identity development models and links between identity development stages and adult attachment theory.” Participants who had not completed the phase two survey received the following question: “On average, participants rated this recommendation as *important*; you did not rate this item. Based on this information, please rate your response to the recommendation below using the 5‐point scale.” Consensus was achieved by using a 75% benchmark (i.e., 75% of participants agreed a guideline was important or very important), as recommended by Diamond et al. (2014).

### Data analysis

#### Phase 1

Thematic analysis (TA; Braun & Clarke, 2006, 2019) was used to analyze data from the first phase of the study. TA is a qualitative method used to identify, analyze, and report patterns (i.e., themes), which is recommended for use in Delphi Studies (Brady, 2015). TA has six phases: (a) Become familiar with the data, (b) generate initial codes, (c) search for themes, (d) review themes, (e) define and name themes, and (f) produce the report. In phase one, participants shared 215 guidelines. The guidelines were downloaded from Qualtrics for analysis and organized by step of EFT into a spreadsheet. Each author familiarized themselves with the guidelines by reading and re‐reading each statement. The authors then met to discuss the data and agreed that there was significant repetition across the 215 guidelines shared by participants in phase one. Next, each author independently reviewed the 215 guidelines in the spreadsheet and generated initial codes in order to reduce the repetition across participant responses as well as to improve conciseness and clarity. The authors met repeatedly over several weeks to discuss the most salient points or potential themes. Using an iterative approach, themes were discussed and refined until consensus was achieved. This process resulted in collapsing the 215 guidelines into 117 guidelines organized into the following themes: therapist foundational knowledge and development, practice set up and orientation, assessment, and adaptations to each step and stage of EFT. The 117 guidelines were then compiled, entered into Qualtrics, and sent to participants to rate their level of importance in phase two.

#### Phase 2

Responses to the second survey were downloaded from Qualtrics into IBM SPSS Statistics (Version 28.0; IBM Corporation, 2022). Each statement was entered as a separate variable in SPSS. Frequencies were then run for each statement. All statements that achieved greater than or equal to 75% consensus (i.e., were rated as important or very important by at least 75% of participants) were immediately accepted (Diamond et al., 2014). This resulted in a total of 106 retained guidelines. The four statements that achieved a consensus level just below the cut‐off for consensus (i.e., 70%–74% of participants rated the statements as important or very important) were extracted to be re‐rated in phase three. Statements that were rated as important or very important by 69% or fewer participants were removed (Diamond et al., 2014). This resulted in removing seven guidelines. In addition, participants recommended three new statements in phase two. Thus, in phase three, participants re‐rated the four statements ranked just below the consensus benchmark as well as rated the three new statements generated by participants in phase two for the first time.

#### Phase 3

All statements were downloaded from Qualtrics. As in the previous phase, descriptive statistics were used to assess which recommendations should be retained using the 75% consensus benchmark. The four guidelines that participants were asked to re‐rate and the three new guidelines provided to participants in phase three were all endorsed as important or very important by 75% of the panelists. Combined with the 106 statements retained in phase two, this resulted in a total of 113 guidelines. Using the TA process described in phase one, the 113 guidelines were then reviewed by all authors to reduce repetition and improve conciseness and clarity. Again, this involved an iterative process of reviewing the statements, meeting to discuss themes, identifying areas of repetition, determining statements that needed to be clarified, organizing statements into themes, and preparing the final list of recommendations. A key priority of this process was to ensure the final statements fully and adequately captured the recommendations shared by participants. This iterative process resulted in 49 final guidelines organized into the following categories: therapist foundational knowledge and development, practice set‐up and orientation, assessment, and each stage and step of EFT.

## RESULTS

### Phase 1

In phase one, participants generated 215 statements, which were collapsed into 117 statements during TA. Participant statements related to the following themes: therapist foundational knowledge and development, practice set up and orientation, assessment, and adaptations to each step and stage of EFT. Participants indicated EFT therapists working with SS/SG relationships should develop additional knowledge and skills prior to providing services. For example, one respondent recommended each therapist should:

Examine [their] own relationship and sexuality biases regarding what makes a “healthy relationship;” what structure[s] you think a relationship should have, how partners should behave, agreements that are required to foster security (especially regarding monogamy, sexual exclusivity and safe sex) and reflect on whether you can be aware of your own biases showing up in the therapy process and can respond flexibly in order to step back from [your] own ideas and fully join with and honor the relationship and the partners you are working with. Take responsibility for learning about relationships and gender expressions different from yours and outside of your realm of experience so that you do not put the burden on the clients to “educate” you.

Additionally, participants suggested therapists should ensure their paperwork was affirming and inclusive of all gender and sexual identities and relationship structures, (e.g., providing fill in the blank options and opportunities to self‐describe). Participants also recommended therapists' websites specifically display LGBTQ+ affirming images and messages (e.g., a progress flag). When conducting assessment, many participants recommended exploring how each partner understood their sexual and gender identities, discussing “coming out” stories and “reparative attachment experiences,” such as those with chosen family, through an attachment history, and overtly exploring how participants are being impacted by current sociopolitical events impacting LGBTQ+ communities (e.g., anti‐trans laws).

Therapists recommended alterations for each step of EFT. When discussing building alliance in step one, one respondent stated, “I try to make explicit that I honor their longings for intimacy, sense of belonging, and for love as…universal.” Another respondent, when discussing step two, indicated that therapists should, “Look for core beliefs about roles, core beliefs about self, [and]…explore how much the person feels they must represent their sexuality/gender in confrontation and/or resolution.” In step three, participants discussed positioning and fully validating unacknowledged emotions within the client's sociocultural context. Additionally, many participants indicated that reframing the cycle as the problem occurring within a hostile sociocultural context was necessary for step four.

In step five, one respondent indicated it is necessary to, “Normalize that they may have split off aspects of self and needs due to not being a part of a heterosexual‐normative world; validate that and help them integrate these parts with empathy and understanding.” This reflected an overall emphasis on taking additional time to re‐integrate parts of self that were disowned due to living in a rejecting society. For example, one participant recommended saying, “You have been left when you've shared your heart/yourself with loved ones before, and so of course this fear is here alive in the room now as I ask you about sharing more of your heart with your person.” Step six, which involves promoting acceptance of the other partner's new experience, was described by one participant as a

…complex task especially when one or both partners are still suffering from the effects of the trauma that so many same gender‐attracted people carry with them into intimate relationships. Building this empathy will greatly assist in the creation of new interactional patterns based on trust and caring.

In addition to increasing mutual empathy, several participants discussed the need to tread carefully because the other partner may not accept their partner's new experience of themselves and their identity, especially if this experience runs counter to how they previously understood themselves.

Step seven, facilitating the expression of needs and wants, may take more time with clients in SS/SG relationships because they may have suppressed their relational wants and needs. Indeed, one expert recommended to:

Notice and name that this is probably very different and perhaps the first time they are attempting this and of course it would be scary. Help them slice it thinner and talk about how perhaps they've never been able to do this and have always been on their own at times like this and don't know how.

In addition to slowing down and increasing the amount of time needed in therapy, participants indicated that finding new solutions to old problems (step eight) may be more difficult, take longer, and may not appear the same as solutions in heterosexual, cisgender (i.e., different‐gender) relationships. Moreover, finding these new solutions involves highlighting “the shifts that have happened for each in regard to their view of self and view of other as members of LGBTQ+ communities and how that may have played a part in their cycle previously, but has now shifted.” Finally, for step nine (i.e., consolidation), a participant explained that therapists should:

Honor the story of the trauma growth, the obstacles they have had to overcome, the fight to be themselves, and to love in the way that feels most meaningful to them…. Reframe how they lost their way and found each other again and how they can now go more safely into the world knowing that they are deeply connected and can flexibly meet life's challenges.

### Phase 2

Of the 117 statements rated by participants in phase two, 106 statements were retained using the 75% consensus benchmark, seven statements were removed, and three new statements were generated. The guidelines that were removed are presented in Table 2.

**TABLE 2:** Guidelines removed after phase 2.

Statement	Phase 2 very important (%)	Phase 2 important (%)	Phase 2 neither important nor unimportant (%)	Phase 2 unimportant (%)	Phase 2 very unimportant (%)
HIV status and whether they are taking precautions.	25.8	28.1	32.3	3.1	9.4
Sexual interests (e.g., top, bottom, versatile) and the impact they can have on the relationship.	22.6	29.0	32.3	9.7	6.5
Be prepared to discuss your own sexual orientation and history with LGBTQ + communities.	25.0	21.9	34.4	12.5	6.3
Have a working knowledge of LGBTQ + communities. Immerse yourself in LGBTQ + communities (i.e., follow LGBTQ + issues in the news, attend community events)	28.1	37.5	34.4	N/A	N/A
Be knowledgeable about LGBTQ + models of identity development (e.g., Cass, Fassinger)	28.1	37.5	31.3	3.1	N/A
Disclose your own gender and sexual identities early on in therapy.	25.8	19.4	45.2	3.2	6.5
Encourage clients to explore other people, services, sports, churches, activities, or groups they may want to connect with.	33.3	30.0	23.3	10.0	3.3

### Phase 3

The four guidelines participants re‐rated and the three new guidelines generated in phase two were all retained using the 75% consensus benchmark. The seven guidelines retained in phase three plus the 106 guidelines retained in phase two resulted in 113 guidelines; using TA, these were collapsed to 49 final guidelines. The final guidelines are shown in Table 3.

**TABLE 3:** Guidelines for working with LGBTQ+ relationships.

Category	Guideline
Prior to and throughout therapy	
Therapist foundational knowledge and development	*Understand minority stress, diverse sexual practices, identity development, relationship structures, identity‐related trauma, and seeking supervision on assumptions, prejudices, and capacity to attune to emotional and attachment messages and needs*
	1. Understand trauma and minority stress, their impact on relationships, and the connections between trauma, minority stress, and adult attachment
	2. Have a basic knowledge of sex therapy, including how to assess sexual history and diverse sexual practices (e.g., BDSM, kink)
	3. Understand LGBTQ+ identity development models and links between identity development stages and adult attachment
	4. Understand diverse relationship structures (e.g., nonmonogamy, power exchange)
	5. Seek supervision to actively work on the therapist's own prejudices, biases, and assumptions, including homophobia, transphobia, heterosexism, cissexism, and mononormativity
	6. Actively integrate cultural humility into therapeutic work, including how to repair with the client when needed
Practice set‐up and orientation	*Determine ways to center LGBTQ+ identities in the practice including on intake forms, marketing materials, and psycho‐education materials*
	7. Assess sexual identity, relationship structures, gender identity, and preferred pronouns on intake forms
	8. Indicate the therapist is LGBTQ+ affirming in marketing materials
	9. Be cautious about recommending books and materials that contain only examples of heterosexual relationships, such as the book *Hold Me Tight*
Assessment	*Explore how each person identifies, who knows about their identities and relationships, sources of resilience, and how minority stress impacts identity and attachment strategies*
	10. Assess each person's identities (e.g., gender identity, sexual identity, religious background, race, ethnicity, ability status)
	11. Explore each person's coming out/attachment history, including who knows how each person in the relationship identifies, who knows about the relationship(s), and the experience of telling others about their identities and relationships
	12. Identify sources of resilience (e.g., have they created their own traditions? How are sexual and gender identities celebrated? What people, places, communities, and organizations, are places they experience as celebrating their relationship(s)?)
	13. Explore the ways in which minority stress has impacted view of self and other, sexual identities, and relationship(s)
	14. Assess for attachment injuries related to identity and how they impact their current relationship(s)
Stage one	
Step one	*Critical aspects of alliance include spending more time in content, using clients' words, acknowledging minority stress, and lateral violence*
	15. Spend more time in content, including discussing clients' experiences of their body, their relationships, the political climate, and intersectional identities
	16. Use clients' words (e.g., partner, wife, dom, kitchen table poly)
	17. Explicitly acknowledge minority stress and current political challenges; note these can contribute to the negative relationship cycle
	18. Understand that intimate relationship violence driven by internalized homophobia and transphobia may occur
Step two	*While identifying the negative cycle, understand that gender, adaptive emotional strategies, and trauma can impact interactional positions*
	19. Understand that gender and gender expression may impact how the cycle is enacted for all partners
	20. Be aware that hiding emotions and identity might be necessary to survive
	21. Validate the impact of past trauma and marginalization in the development and exploration of attachment longings and emotions When tracking the cycle, incorporate what is learned in assessment regarding trauma history, experiences of minority stress, identity development, rejection from family of origin, and lived experiences in the SS/SG relationship
	22. Reframe both positive and negative forms of coping as protection from a hostile sociocultural environment
	23. Ask for consent to slowly access somatic experiences and emotions (e.g., rather than ‘what's happening in your body,’ ‘I notice that your fist clenched when you shared that experience. Would it be okay if we visited what just happened with your hand?’)
Step three	*While accessing underlying attachment emotions, normalize the impact of marginalization and make relevant unhealed wounds explicit*
	24. Normalize feelings of rage, fears of replicating the harm done to them, fears of their partner(s) replicating the harm done to them, and reluctance to be vulnerable; honor how it feels to have others hold and validate those feelings when they have often held them alone
	25. Make explicit any unhealed wounds related to sexual and gender identity that block access to connection in the present
	26. Be aware of how gender socialization impacts the client's ability to access and express emotion
Step four	*Reframing the problem as the cycle includes a systemic understanding that interactional cycles are impacted by sociocultural contexts and stigma*
	27. Be careful not to use language that presupposes the only barrier to feeling secure is how clients engage with their partner(s); highlight how stigma, instability, and the fight to create the life they deserve also erodes safety
	28. Link view of self and other to clients' sociocultural contexts (e.g., minority stress, internalized cisgenderism, homophobia, and heterosexism) and their action tendencies in their relationship(s)
	29. Ensure reframing the cycle includes clients' sociocultural contexts (e.g., minority stress, internalized cisgenderism, homophobia, and heterosexism)
	30. Validate the significant attachment trauma they endured because of their identity and allow more time and support to reach new experiences of safety
Stage two	
Step five	*While accessing implicit needs, fears, and models of self may involve working with embedded internalized homo−/trans‐phobia and moving from shame to acceptance to pride*
	31. Help each person hear and tolerate the other's negative view of self and other as it relates to their family of origin and sociocultural context (e.g., minority stress, internalized cisgenderism, homophobia, and heterosexism)
	32. Acknowledge that clients who've experienced identity‐related trauma need more time and support to feel safe identifying disowned needs and aspects of self
	33. Invite deeply embedded internalized homophobia, transphobia, cissexism, and heterosexism into the conversation, such as when a partner's attachment fears are stoked by a partner's changing identity or fears of a partner not being comfortable with their own identity
	34. Be aware that promoting identification with disowned parts of self may involve the client shifting from feeling shame to acceptance to pride
Step six	*Promoting acceptance of partner(s) fears and models of self requires acceptance of each person's identity, discussion about where expressing vulnerability is safe, and negotiating which aspects of models of self are worked with in relationship therapy versus individual therapy*
	35. Recognize, normalize, and validate each partner's experience of their sexual and/or gender identity and facilitate acceptance by the other
	36. Acknowledge that this new way of interacting may feel unsafe in some settings so it may help to share the settings where they feel safe and have a plan for how to stay connected where they feel unsafe
	37. Understand and validate how clients may distance themselves from their partner based on their internalized homophobia, transphobia, heterosexism, and cissexism. Validate clients' needs to feel safe and challenge dismissive statements made by their partner(s)
	38. Consider that acknowledging disowned needs and aspects of self can activate blocks in the partner(s) because they have previously felt united against stigma and some of their partners' longings may feel like a threat or a judgment of them
	39. Be aware that not all feelings should be shared since some could be actively damaging (e.g., a nontrans partner may need space to grieve, adjust, and seek support outside of the relationship because sharing that with the trans‐person who is actively transitioning their identity could be damaging)
Step seven	*Naming and asking for needs to be met is hindered by years of sociocultural challenges, which requires the therapist to validate their legitimacy*
	40. Recognize that shame around sexual and/or gender identity may make it difficult to recognize one's needs and wants, especially within hostile sociopolitical and sociocultural contexts
	41. Validate that clients deserve loving, long‐lasting relationship(s)
	42. Help clients feel their needs and wants are legitimate and are valid to express without fear or shame
Stage three	
Step eight	*While facilitating new solutions, it is important to acknowledge that some contexts will require those in relationships to focus on safety and protection instead of sharing more vulnerably*
	43. Acknowledge that the openness experienced in therapy may be difficult to maintain in unsafe public environments
	44. Explore the barriers to finding new solutions to relational problems in SS/SG relationships
	45. Highlight the shifts that have happened for each client regarding their view of self and others as members of LGBTQ+ communities and how that previously played a role in their cycle
Step nine	*Consolidating new, positive cycles offers an opportunity to name that they can be a shelter for each other during challenging times and that they are charting territory with few role models; be sure to explore a variety of resources that reflect and validate their relationship(s)*
	46. Acknowledge that they are each other's shelter not only in the normal storms of life but in a world that is often unsupportive and harsh to SS/SG relationships
	47. Contextualize their growth within a place of less privilege and having fewer models and scripts than people in heterosexual relationships
	48. Explore new ways of interacting with family and friends as well as ways of accessing community support and chosen family connections to ensure clients are well resourced
	49. Acknowledge that their work is a radical act of resistance to societal messages and validate their achievements

## DISCUSSION

The purpose of this study was to identify guidelines for using EFT with SS/SG relationships based on expert consensus using the Delphi method. Although research documents EFT therapists work with SS/SG relationships (Allan et al., 2022); to date, all application of EFT with SS/SG relationships has been conceptual (Allan & Johnson, 2017; Hardtke et al., 2010). This paper, therefore, presents the first formal documentation of therapists' recommendations for the use of EFT with SS/SG relationships.

Research continues to demonstrate that culturally adapted psychotherapy is more effective than nonadapted psychotherapy (Nagayama‐Hall et al., 2016; Soto et al., 2018). Culturally adapted psychotherapy that aligns with client worldviews and cultural background has been found to increase client engagement, decrease client dropout, and result in better clinical outcomes (Nagayama‐Hall et al., 2016; Soto et al., 2018). Furthermore, members of LGBTQ+ communities prefer clinical treatment that addresses client sociocultural context, identity, and presenting concerns related to these experiences (Berke et al., 2016; Goldbach & Holleran Steiker, 2011; Pentel et al., 2021; Scott & Rhoades, 2014). Therefore, it is incumbent on EFT therapists to address these concerns when working with SS/SG relationships.

Data gathered in this study identified initial guidelines for the adaptation of the three stages and nine steps in the EFT model, while also recognizing additional preparation needed before initiating therapy, including guidelines for therapist foundational knowledge and development, practice set‐up and orientation, and assessment. Regarding therapist foundational knowledge, participants noted the importance of understanding minority stress and the impact it has on SS/SG individuals and relationships. Expert panelists indicated that both distal and proximal stressors may impact a client's internal working models of self and others and how clients approach and experience intimate relationships, thus necessitating the adaptation of EFT. The expert panel also noted the importance of sex therapy training and being aware of diverse relationship structures (e.g., not assuming monogamy as ideal or desired).

A second area of identified importance was therapist development. Like all effective therapy practices (e.g., Zhang et al., 2021), demonstrating cultural humility and seeking consultation on less familiar topics or populations was recommended. Expert panelists also indicated that understanding how marginalization may be experienced as traumatic and trauma‐informed practices may be necessary when working with SS/SG relationships. The third area identified was practice set‐up and orientation. Participants described the importance of affirming that clients are welcome to present and explore their identities and experiences. Additionally, expert panelists identified that traditional ways of conducting assessment in EFT should be expanded to explore sexual, gender, and racial/ethnic identities, among others; this may include discussing how clients have explored their identities and where and with whom they feel safe to engage in identity exploration.

The participants also made recommendations for each stage of EFT. In stage one, integrating an awareness of and exploring the impact of discrimination and stigma or minority stress was recommended. This aligns with existing literature suggesting that expressive flexibility is an important stigma coping resource (Wang et al., 2022). Furthermore, panelists indicated that the impact of stigma, discrimination, and minority stress should be integrated into the negative interaction cycle to explore how these experiences perpetuate relational distress. Participants also recommended to explore the impact of distal and proximal stressors and identity development on internal working model(s) of self and other(s). Finally, therapists should recognize the defenses required to cope with stigma and discrimination as important survival mechanisms that are required by people in SS/SG relationships and the need to continue to use them to protect themselves throughout life. In other words, they are not therapeutic *blocks* or *defenses* but rather are important adaptive strategies needed to survive.

Participants noted that stage two work in EFT is emotionally deeper and more expressive and requires continued care to integrate *disowned aspects* of identity as well as the vulnerable aspects of the negative interaction cycle noted in EFT. Hiding one's true identity is an important survival strategy for some in SS/SG relationships and integrating the impact of this concealment in stage two is part of the adaptations suggested. This may involve supporting identity development where clients move from feeling shame regarding their identities to feeling proud of them. Another recommendation for stage two was to recognize that asking for and expecting an affirmative response to one's emotional needs may be new for some and that it is important to affirm that their needs and wants are legitimate and valid to express without fear or shame.

Finally, in stage three, panelists reflected that the relationship can be an important source of resilience for those in SS/SG relationships. This aligns with previous literature indicating romantic relationships are inherent to individual wellbeing as well as overall health (MacIntosh & Butters, 2014). In stage three, additional, more explicit discussion of resources and connections is needed to support SS/SG romantic relationships, which aligns with the cultural adaption literature (e.g., Nagayama‐Hall et al., 2019). This can include community resources or (chosen) family that recognize and affirm their relationships.

While the generated guidelines broadly discuss the adaptations needed for working with SS/SG relationships, each unique relationship structure (e.g., lesbian couples, trans/nonbinary couples), requires specific adaptations to respond to their unique needs and lived experiences. For example, transgender and gender nonconforming (TGNC) couples may need therapy that overtly addresses gender minority stress (Testa et al., 2015) and expands ideas of how emotional avoidance and suppression are contextualized within gender essentialism (see Edwards, Wittenborn, et al., n.d.). EFT with gay male couples may involve overtly addressing relational ambiguity (Green & Mitchell, 2015), while EFT with lesbian couples may require therapists to address how and when clients are sharing their attachment emotions, needs, and longings (Hardtke et al., 2010; Scott & Rhoades, 2014). Further research is needed to explore how EFT should be adapted to each unique relationship structure.

## IMPLICATIONS

There are several implications of these results for EFT practitioners. They include the need to pursue additional training and ongoing supervision/consultation, explore one's own sexual and gender identity, and develop an awareness and understanding of the impact of minority stress including the ability to track both the intrapsychic and interpersonal implications of minority stress, link proximal and distal stressors with internal working models of self and others, and develop an understanding of how experiences of stigma and discrimination impact emotional expression and suppression strategies. Developing these skills may require deliberate practice as therapists develop the knowledge and awareness to deliver EFT in a way that fits the needs of SS/SG relationships. These recommendations align with Chow et al.'s (2015) findings regarding deliberate practice and SAMHSA's (2016) guidelines for working with marginalized groups.

While some literature (e.g., Gottman et al., 2003; Kurdek, 2004) treats SS/SG relationships and heterosexual, cisgender (e.g., different‐gender) relationships as similar, several guidelines (e.g., American Psychological Association (APA) Task Force on Psychological Practice with Sexual and Gender Minority Persons, 2021; Goldberg & Allen, 2013) have described the need for interventions tailored to the unique needs of SS/SG relationships. Many of these recommendations align with the guidelines for adapting EFT proposed by the expert panel. For example, both Scott and Rhoades (2014) and Green and Mitchell (2015) discuss the need for therapists to be aware of and directly address minority stress as well as help clients explore and access community support. Furthermore, Pentel et al. (2021), in the only study that systematically adapted an evidence‐based treatment (i.e., Cognitive Behavioral Couple Therapy) for female SS/SG couples, delineated modules such as discrimination, outness and disclosure, and family of origin—all of which align with the EFT guidelines generated in the current study. Additionally, Whitton et al. (2018) outlined guidelines for providing relationship education programs to SS/SG couples; these recommendations include the removal of heterosexist bias, tailoring core content to common SS/SG couple experiences, providing relevant novel content, and addressing clinician bias. The APA guidelines on working with sexual and gender minorities recommend trauma‐informed care and Brown (2013) discusses working with both nonmonogamous relationships and internalized shame in gay male relationship therapy.

## LIMITATIONS

The psychotherapy literature on SS/SG relationships often neglects issues of race, class, and gender (Addison & Coolhart, 2015), and this is partially echoed in our findings. The panel addressed how gender impacts emotional expression and the negative interaction cycle; however, there was limited discussion of race and class. SS/SG relationships are more likely to be interracial and composed of individuals from different classes (Gates, 2014). Therefore, these are important aspects to address in future research on EFT with SS/SG relationships.

Similar to other studies (e.g., Mohr et al., 2013), a variety of identities and relationship structures were subsumed under the label of SS/SG relationship, limiting our ability to explore in depth the ways therapists adapt EFT when working with individuals in relationships who have different gender and sexual orientation identities. Further, literature on the Delphi method indicates a response rate of at least 70% ensures trustworthiness. While our average response rate across all phases in the study was 70%, it fell to 67.5% in phase two.

In addition, while all participants met the inclusion criteria, many respondents (46.15%) reported that their work with SS/SG relationships only comprised 5%–10% of their caseloads. Results may have been improved if we had identified and engaged more participants with larger caseloads of SS/SG relationships and personal lived experience in SS/SG relationships, as research suggests clients who identify as LGBTQ+ benefit from therapists who match their identity (Jones et al., 2003).

The self‐ratings for both EFT expertise and expertise in SS/SG relationships were lower than expected. It is possible that because EFT is a challenging model to learn (Allan et al., 2016), participants do not feel as though they have fully mastered the model. Furthermore, it is possible that even though this panel may have specific expertise with SS/SG relationships, cultural humility prevents them from calling themselves experts and/or they may still believe they have much to learn, as the literature on SS/SG relationships is scarce. Therefore, these results should be considered preliminary and future research is needed to continue to identify best practices for adapting EFT for SS/SG relationships to ensure that harmful stereotypes and folklore are not perpetuated.

## CONCLUSION AND FUTURE RESEARCH

EFT is rooted in attachment science, which has been applied across relationships and cultures. Yet, research indicates attachment experiences may be different across sociocultural contexts, necessitating the adaptation of evidence‐based models when applying them to marginalized populations. To add to the cultural adaptation literature, this article presented the first systematic study of adaptations made to EFT for SS/SG relationships by expert EFT therapists. These guidelines indicate EFT therapists working with SS/SG relationships should take additional steps, such as additional trainings and supervision, as well as integrating additional ways of practicing, to successfully work with SS/SG relationships. Furthermore, these guidelines demonstrate that therapists working with SS/SG relationships believe modifications to EFT should occur throughout the process of therapy, from modifying assessment questions to the integration of client identity and sociocultural context in stage two of EFT. By modifying EFT to account for both client identity and sociocultural context, EFT therapists can provide culturally responsive care for SS/SG clients facing marginalization and minority stress. Future research should focus on providing in depth, qualitative analysis of the use of EFT with SS/SG relationships and LGBTQ + relationships more broadly, as ongoing understandings of this topic and iterations of research will both deepen and broaden understandings of EFT with marginalized populations.
